# Clinical Outcomes of Arthroscopic Adhesiolysis: A Case Series of 40 Patients With Postoperative Knee Stiffness

**DOI:** 10.7759/cureus.63378

**Published:** 2024-06-28

**Authors:** Vikram Sapre, Yash Dhanwani, Navneet Saluja, Ankit M Jaiswal, Rohan Chandanwale

**Affiliations:** 1 Orthopedics, N.K.P. Salve Institute of Medical Science and Research Center and Lata Mangeshkar Hospital, Nagpur, IND; 2 Orthopedics and Traumatology, Jawaharlal Nehru Medical College, Datta Meghe Institute of Higher Education and Research, Wardha, IND; 3 Orthopedics, Mata Gujri Memorial Medical College, Kishanganj, IND; 4 Orthopedics, Jawaharlal Nehru Medical College, Datta Meghe Institute of Higher Education and Research, Wardha, IND

**Keywords:** knee rom, arthroscopic release, postoperative knee stiffness, adhesiolysis, arthrofibrosis

## Abstract

Introduction

Restricted range of motion over the knee joint is a known complication following the surgical procedure. Aggressive rehabilitation protocols can initially manage knee stiffness due to arthrofibrosis. If conservative management fails, surgical (open or arthroscopic) release is the preferred modality of management. We present a series of 40 patients with postoperative knee stiffness who were treated with arthroscopic adhesiolysis.

Material and methods

This is a retrospective study conducted at Phoenix Orthopedic Superspeciality Hospital, Nagpur, India, from 2017 to 2021. Our study included 40 patients with postoperative knee joint stiffness, of whom 27 were males and 13 were females. The study considered the duration of stiffness, which ranged from six months to five years. All patients underwent arthroscopic knee release. A rigorously supervised physical therapy program followed this procedure. Patients were examined at three months, six months, and one year to assess improvement in knee range of movement.

Results

Out of 40 patients, six were classified as Shelbourne type 4, and the remaining were Shelbourne type 3. Twenty-three of 40 patients developed arthrofibrosis following intra-articular or peri-articular fracture fixation surgery; 11 patients were operated on arthroscopically for anterior cruciate ligament (ACL) or posterior cruciate ligament (PCL) reconstruction. Three patients developed stiffness following total knee replacement, one following patellectomy, and three following infection after fracture fixation. The mean pre-op knee range of motion (ROM) was 48.875 degrees. Following arthroscopic release, the mean improvement in ROM was 60 degrees intra-operatively. The average postoperative range was 108.25 degrees.

Conclusion

Arthroscopic adhesiolysis and quadriceps release are reliable methods for dealing with postoperative knee stiffness. It prevents wound complications and increases the chances of surgical site infection due to smaller incisions. Postoperatively, we achieved an average increase of 60 degrees in ROM over the knee joint.

## Introduction

Restricted range of motion (ROM) over the knee joint is a known complication following the surgical procedure. Surgeries procedures include anterior cruciate ligament (ACL) and posterior cruciate ligament (PCL) reconstruction, total knee arthroplasty, open reduction internal fixation (ORIF) of the fractures near the knee joint, infections, and open fractures. Most cases of knee stiffness occur due to faulty surgical procedures and non-compliance with postoperative rehabilitation [1]. Knee stiffness in total knee arthroplasty can occur due to prior knee surgery, incorrect positioning, over-sizing, incorrect gap balancing, arthrofibrosis, complex regional pain syndrome, infection, and heterotopic ossification. Knee stiffness due to arthrofibrosis can initially be managed with aggressive rehabilitation protocols. If conservative management fails, surgical (open or arthroscopic) release is the preferred modality of management. Careful selection of the patient is the key to the successful outcome of adhesiolysis [2]. Arthrofibrosis is also common after ACL reconstruction surgery. Suboptimal rehabilitation following the ACL reconstruction is the most important factor leading to arthrofibrosis [3,4]. The timing of surgery following the ACL injury also influences the chances of arthrofibrosis. Shelbourne et al. concluded in their study that patients who underwent the ligament reconstruction during the first week after trauma (Group I) had a significantly increased incidence of arthrofibrosis in comparison to patients with ACL reconstruction delayed for three weeks or more (Group III). Patients who underwent the surgery between one and three weeks (Group II) had similar chances of arthrofibrosis to Group I patients [5]. An accelerated rehabilitation to achieve full knee ROM within six weeks following the ACL reconstruction is effective in preventing arthrofibrosis. Postoperative restriction of knee ROM is also a problem following the open reduction of internal fixation for compound or closed fractures around the knee joint. There is a significant loss of bending over the knee joint following infective arthritis or osteomyelitis around the knee joint.

The primary reason for restricted knee ROM in arthrofibrosis is the formation of fibrous adhesion inside the knee and between the anterior aspect of the femur and quadriceps mechanism. Release of such adhesions either with the open or arthroscopic procedure generally leads to significant improvement in the ROM [6-8]. Open adhesiolysis is generally associated with a higher chance of infection, wound complications, and larger scars. Also, open release of adhesion requires immobilization, which is against early rehabilitation to regain the ROM. Liu KM et al. proposed a minimally invasive method for the release of both intra-articular and extra-articular adhesions and found favorable clinical outcomes [9]. A careful selection of patients depending on their history and pre-operative assessment can significantly avoid complications and lead to good clinical outcomes. Shelbourne et al. (1996) proposed a classification of patients with arthrofibrosis based on their pre-operative profile [10]. We present a series of 40 patients with postoperative knee stiffness who were treated with arthroscopic adhesiolysis. We included patients with Shelbourne types 3 and 4.

## Materials and methods

This is a retrospective study conducted at Phoenix Orthopedic Superspeciality Hospital, Nagpur, India, from 2017 to 2021. Our study included 40 patients with postoperative knee joint stiffness, of whom 27 were males and 13 were females. Patients ranging in age from 19-54 years were included in the study. The duration of stiffness, from six months to five years, was considered for the study. A thorough pre-operative evaluation was done with operative history, current ROM, amount of deficit, scars, skin condition, and quadriceps function. Patients were classified according to the classification proposed by Shelburne et al., depending on their pre-operative patient profile. Patients with active infection, any malunion or deformity, articular incongruity visible on x-rays, and more than 20 degrees of fixed flexion deformity were excluded from the study. All patients underwent arthroscopic knee release. A rigorously supervised physical therapy program ensued. Patients were examined at three months, six months, and one year to assess improvement in knee ROM.

Surgical technique

The surgical procedure was performed under combined spinal epidural anesthesia. ROM was evaluated under anesthesia, and a thigh tourniquet was applied. After routine sterile preparation, anterolateral and anteromedial portals were created. The anterolateral portal was used as a visualization portal, and the anteromedial portal was used as an operating portal. Thorough debridement of the joint with excision of fibrous bands, nodules, and adhesions was done from the supra-patellar pouch, medial and lateral gutters, release of the infra-patellar fat pad, and medial and lateral compartments, along with gentle manipulation. After the intra-articular release of the adhesions, a perforation was made in the supra-patellar pouch to release the adhesion between the quadriceps mechanism and the anterior femur. Adhesion between the implant and the quadriceps mechanism was released. Medial supra-patellar and lateral supra-patellar portals were made to release the extra-articular adhesions further proximally. The periosteum elevator was used to release the adhesions on the anterolateral aspect of the femur. Each step of release was followed by manipulation under anesthesia to see for improvement in the ROM and the location of further adhesions. If required, a mini-open incision was given on the lateral side to perform the open release of adhesions. Once the adequate release was done, the wounds were closed over the negative suction drain. Intra-operative ROM achieved was noted after the final release. Patients were given a hinged knee brace for one week, with weight bearing as tolerated. Epiduaral analgesia was continued for 48 hours in all patients. Early and aggressive physiotherapy was advised for all patients. All patients received a supervised physical therapy program postoperatively.

## Results

The study included 40 patients, of whom 27 were male and 13 were female. Six patients were classified as Shelbourne type 4, and the remaining were Shelbourne type 3. Twenty-three of 40 patients developed arthrofibrosis following intra-articular or peri-articular fracture fixation surgery; 11 patients were operated on arthroscopically for anterior cruciate ligament reconstruction or posterior cruciate ligament reconstruction. Three patients developed stiffness following total knee replacement, one following patellectomy, and three following infection after fracture fixation (Table 1).

**Table 1:** Distribution of patients according to the cause of knee stiffness.

Pre-operative history	Number of patients
Fracture fixation	23
Arthroscopic procedures (anterior cruciate ligament reconstruction/posterior cruciate ligament reconstruction)	11
Total knee replacement	2
Infection	3
Patellectomy	1
Total	40

Twenty-four patients had stiffness for less than one year, while 16 patients had stiffness for one year or more. We followed the patients at three months, six months, and one year postoperatively. The mean pre-op knee ROM was 48.875 degrees. Following arthroscopic release, the mean improvement in ROM was 60 degrees intra-operatively. The average post-op range was 108.25 degrees. It was also noted that the improvement in ROM was closely related to the duration of stiffness, which may be due to the development of extra-articular adhesions. Improvement in the ROM was also directly related to the cause of arthrofibrosis as well as the presence or absence of infection. Maximum improvement was observed in patients who were operated on within one year of the onset of stiffness. The mean improvement in ROM at the final follow-up was higher in patients with less than one year of duration of stiffness than in patients with more than one year of duration of stiffness. In patients with more than a year of history of stiffness, the mean improvement in ROM intra-operatively was 59.375, and in patients with less than a year of history of stiffness, the mean improvement in ROM was 61.04. Also, patients who had stiffness following an arthroscopic procedure showed a better outcome than any other cause of arthrofibrosis. Three complications that we encountered were an extension lag of 30 degrees in one patient, which recovered to 10 degrees with physiotherapy. One of the patients had severe discharge, which settled with antibiotics, and one had a history of recurrent patellar subluxation following the arthroscopic release. This was treated with a medial patellofemoral ligament reconstruction. We had no cases of iatrogenic fractures during the intra-operative manipulation under anesthesia.

## Discussion

Arthrofibrosis is a seriously debilitating condition leading to loss of extension in the early stages, followed by flexion. Prevention is the best form of treatment to manage this serious complication following any surgical intervention. In most cases, recognized at an early stage, the mainstay of treatment is physical therapy. Adequate supervised physical therapy also helps in the maintenance of muscle strength, which is lost secondary to disuse. In neglected or resistant cases, surgical intervention is required. It is imperative to ascertain the time and nature of the procedure to be carried out [11]. Forced manipulation may result in undue soft tissue injury, further scarring, chondral damage, patellar tendon rupture, and even fractures. Traditionally, arthrofibrosis was managed by open release, which was associated with complications like infection, inadequate release, recurrence, and extension lag [12].

The arthroscopic release offers a minimally invasive procedure with a good to excellent outcome in cases of arthrofibrosis. It stands out as a safer, more reliable, and more predictable procedure, with significantly less damage to already scarred tissues and reduced postoperative pain, leading to faster rehabilitation [13]. Further arthroscopic procedures also reduce the chances of infection and permit a direct evaluation of the condition of the cartilage. Traditionally, open adhesiolysis has been carried out to improve ROM by releasing scarred and contracted quadriceps. This often results in extensive exposure, soft tissue trauma, recurrent adhesion, permanent extensor lag, and infections [14-16]. A minimally invasive arthroscopic adhesiolysis carried out in a controlled and sequential manner further helps improve ROM while avoiding the complications of open release [17-19]. The results of this study reflect that the prognosis following surgical intervention depends on the following factors: duration of stiffness of patients who underwent release within a shorter period of onset attains a better range as compared to long-standing cases; cause of arthrofibrosis for patients with arthrofibrosis following arthroscopic procedures has a better outcome as compared to those following infection; grade of arthrofibrosis.

Patients with arthrofibrosis also have a loss of flexion, which follows extension loss. This may be due to extra-articular as well as intra-articular factors. In this procedure, through the same key-hole incisions, assessment as well as management of these intra-articular pathologies can be carried out. Regarding the timing of adhesiolysis, Cosgarea et al. reported that flexion/extension gains and functional outcomes were satisfactory when the procedure was performed within six months after the prior surgery. Delay or neglect of knee stiffness may lead to the development of extra-articular contractures and adhesions, which may not be amenable to arthroscopic surgery. In our study, the outcomes of adhesiolysis were better when the procedure was performed up to one year after the injury, which is because quadriceps femoris muscle shortening resulting from contracture of the muscle worsens over time after arthrofibrosis. Kim et al. published results of arthroscopic lysis of adhesions and found that among post-traumatic cases (43 of 68 total patients), an increase in ROM was seen from an average of about 70 pre-operatively to 118 postoperatively at a mean final follow-up of 17.8 months [20]. Gittings et al. published the results of arthroscopic lysis of adhesions in post-traumatic stiff knees treated with internal fixation. They found post-lysis ROM improvement from an average of 72 degrees immediately before surgery to 128 degrees intra-operatively (78% improvement) and a sustained clinical improvement to a mean ROM of 101 (35% improvement) at the final follow-up [21]. In the current study, we followed up with the patient for up to one year, and the mean improvement in ROM of the knee joint was found to be 64.37%.

This study had a few limitations. Patients in the study did not belong to a particular age group. Also, the etiological factor for the knee stiffness was varied. This is an important patient factor that affected the mean increase in the knee ROM. Also, patients did their postoperative rehabilitation at different physiotherapy centers according to their convenience. There was no formulated rehabilitation plan for patients undergoing arthroscopic adhesiolysis. This could have affected the results to some extent.

## Conclusions

Arthroscopic adhesiolysis and quadriceps release present a safer, more reliable, and more predictable procedure with faster rehabilitation and better outcomes as compared to open procedures. Arthroscopic adhesiolysis avoids complications like infections, wound dehiscence, and postoperative pain compared to open adhesiolysis. Intra-articular adhesions can be better released with arthroscopic means compared to open adhesiolysis. The authors recommend this procedure for high-grade arthrofibrosis of long duration with no or minimal improvement despite physical therapy.
